# Supplementary material for: Antimicrobial Resistance in Loggerhead Sea Turtles (Caretta caretta): A Comparison between Clinical and Commensal Bacterial Isolates
Source: Animals (Basel). 2021 Aug 18;11(8):2435. doi: 10.3390/ani11082435 (PMC8388645; doi:10.3390/ani11082435)
Supplement: Supplementary file 1 [file animals-11-02435-s001.zip › animals-1330712-supplementary.pdf]

Supplementary materials:

Table S1. Details of type of wounds in 102 injured turtles

Identification number	Age classification	Type of wound
1	Juvenile	Skin wound
2	Adult	Skin wound
3	Sub-adult	Skin wound
4	Juvenile	BAL*
5	Sub-adult	BAL
6	Juvenile	Skin wound
7	Juvenile	Skin wound
8	Sub-adult	Skin wound
9	Sub-adult	Skin wound
10	Adult	BAL
11	Adult	BAL
12	Sub-adult	BAL
13	Juvenile	Skin wound
14	Sub-adult	Skin wound
15	Juvenile	BAL
16	Sub-adult	Skin wound
17	Sub-adult	BAL
18	Sub-adult	BAL
19	Juvenile	BAL
20	Adult	BAL
21	Sub-adult	BAL
22	Sub-adult	Internal biopsies
23	Adult	Skin wound
24	Sub-adult	Skin wound
25	Juvenile	BAL
26	Juvenile	Internal biopsies
27	Juvenile	BAL
28	Juvenile	BAL
29	Adult	Skin wound
30	Sub-adult	Carapace wound
31	Sub-adult	Skin wound
32	Sub-adult	BAL
33	Juvenile	Carapace wound
34	Juvenile	BAL
35	Juvenile	BAL
36	Adult	Skin wound

37	Juvenile	Skin wound
38	Sub-adult	Internal biopsies
39	Juvenile	Internal biopsies
40	Sub-adult	BAL
41	Juvenile	Skin wound
42	Sub-adult	Carapace wound
43	Juvenile	Carapace wound
44	Sub-adult	Skin wound
45	Adult	Internal biopsies
46	Sub-adult	BAL
47	Adult	Skin wound
48	Adult	BAL
49	Sub-adult	BAL
50	Sub-adult	BAL
51	Sub-adult	Skin wound
52	Juvenile	Carapace wound
53	Sub-adult	Carapace wound
54	Sub-adult	Skin wound
55	Juvenile	Carapace wound
56	Sub-adult	Skin wound
57	Sub-adult	Plastron wound
58	Sub-adult	BAL
59	Sub-adult	BAL
60	Adult	Internal biopsies
61	Adult	Skin wound
62	Sub-adult	Internal biopsies
63	Sub-adult	BAL
64	Sub-adult	BAL
65	Juvenile	BAL
66	Sub-adult	Carapace wound
67	Sub-adult	Skin wound
68	Sub-adult	Skin wound
69	Juvenile	Skin wound
70	Sub-adult	Skin wound
71	Adult	Skin wound
72	Sub-adult	Skin wound
73	Sub-adult	Internal biopsies
74	Juvenile	BAL
75	Sub-adult	Skin wound
76	Sub-adult	Carapace wound
77	Juvenile	Carapace wound
78	Juvenile	Skin wound

79	Juvenile	Skin wound
80	Sub-adult	BAL
81	Sub-adult	Internal biopsies
82	Adult	BAL
83	Sub-adult	Skin wound
84	Sub-adult	Skin wound
85	Sub-adult	Internal biopsies
86	Juvenile	Internal biopsies
87	Sub-adult	BAL
88	Sub-adult	Carapace wound
89	Adult	BAL
90	Sub-adult	Carapace wound
91	Juvenile	Skin wound
92	Sub-adult	Skin wound
93	Sub-adult	Skin wound
94	Juvenile	Skin wound
95	Sub-adult	BAL
96	Adult	BAL
97	Adult	Skin wound
98	Adult	Skin wound
99	Juvenile	Skin wound
100	Sub-adult	Carapace wound
101	Adult	BAL
102	Sub-adult	Skin wound

* BAL= bronchioalveolar lavage

Table S2. Details of 16S rRNA gene PCR

<table>
<tr><th>Gene Target</th><th>Primer</th><th>Primer sequence</th><th>Tm (°C)</th><th>Cycling conditions</th><th>Reaction conditions (50 µl)</th></tr>
<tr><td>16S rRNA</td><td>BV5-forward</td><td>(5′-ATTAGATACCCYGGTAGTCC-3′)</td><td>55 °C</td><td rowspan="2">95 °C (10 min)

<u>35 cycles x</u>
94 °C (30 s)
57 °C (30 s)
72 °C (30 s)

72 °C (8 min)</td><td rowspan="2">50 ng template DNA
50 mM KCl
10 mM Tris-HCl pH 8.3
1.5 mM Mg2+
0.2 mM dNTPs
40 pmol of each primer
5U of Taq DNA polymerase*</td></tr>
<tr><td>16S rRNA</td><td>AV6-reverse</td><td>(5′-ACGAGTGACGACARCCATG-3′)</td><td>69.8 °C</td></tr>
</table>

*(AmpliTaq Gold™, Thermofisher, Italy).
